# Rapid Hip Osteoarthritis Development in a Patient with Anterior Acetabular Cyst with Sagittal Alignment Change

**DOI:** 10.1155/2014/523426

**Published:** 2014-10-27

**Authors:** Yasuhiro Homma, Tomonori Baba, Nobuhiko Sumiyoshi, Hironori Ochi, Hideo Kobayashi, Mikio Matsumoto, Takahito Yuasa, Kazuo Kaneko

**Affiliations:** Department of Orthopaedic Surgery, Juntendo University, 2-1-1 Hongo, Bunkyo-ku, Tokyo 113-0033, Japan

## Abstract

Rapidly destructive coxarthrosis (RDC) is rare and develops unusual clinical course. Recent studies suggest multiple possible mechanisms of the development of RDC. However the exact mechanism of RDC is still not clear. The difficulty of the study on RDC is attributed to its rareness and the fact that the data before the onset of RDC is normally unavailable. In this report, we presented the patient having the radiographic data before the onset who had rapid osteoarthritis (OA) development after contralateral THA, which meets the current criteria of RDC. We thought that the increased posterior tilt of the pelvis after THA reinforced the stress concentration at pre-existed anterior acetabular cyst, thereby the destruction of the cyst was occurred. As a result the rapid OA was developed. We think that there is the case of rapid osteoarthritis developing due to alternating load concentration by posterior pelvic tilt on preexisting anterior acetabular cyst such as our patient among the cases diagnosed as RDC without any identifiable etiology. The recognition of sagittal alignment changes and anterior acetabular cyst may play important role in prediction and prevention of the rapid hip osteoarthritis development similar to RDC.

## 1. Introduction

Rapidly destructive coxarthrosis (RDC) was firstly described by Postel and Kerboull in 1970 [1]. It has been characterized as rapid joint destruction within 6 to 12 months, leading to striking joint deformity. Recent studies suggest multiple pathogenesis in rapid hip osteoarthritis development including abnormal cell population in the joint fluid [2, 3] and subchondral insufficiency fracture (SIF) [4]. But the exact mechanism of RDC is still not clear. The difficulty of the study on RDC is attributed to its rareness and the fact that the data before the onset of RDC is normally unavailable. In this paper, we present a case of rapid osteoarthritis development having the radiographic data before and after the onset and discuss the influence of the anterior acetabular cyst and sagittal alignment changes on rapid osteoarthritis developing in the hip.

## 2. Case History

An 80-year-old female had a total hip arthroplasty (THA) on her right hip due to chronic osteoarthritis of the hip for 7 years. At the time of right THA, she had no symptoms on her left hip. However, 2 months after right THA, she started to have left hip pain without any trauma. Gradually left hip pain was deteriorated, as a result left THA was performed 10 months after right THA. After right THA, the patient had no minor trauma such as falls or standing firmly. There were no clinical signs and results of blood test indicating the systematic autoimmune disorder and the infection.

**Figure 1:**
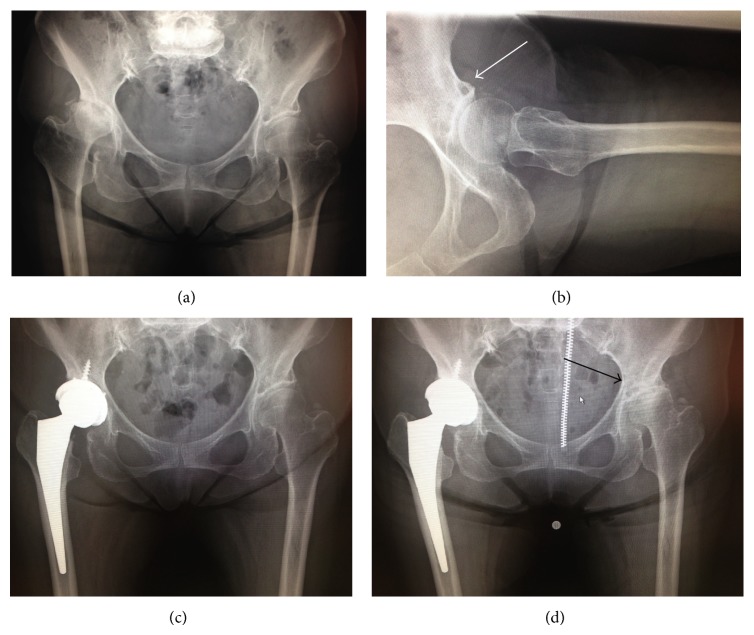
Consequent X-ray of the hip. At the time of right THA, left hip had enough joint space with bone cyst at anterior acetabulum ((a), (b); white arrow). Postoperative X-ray (c). 8 months after right THA, left hip had the end stage of osteoarthritis (black arrow; (d)).

**Figure 2:**
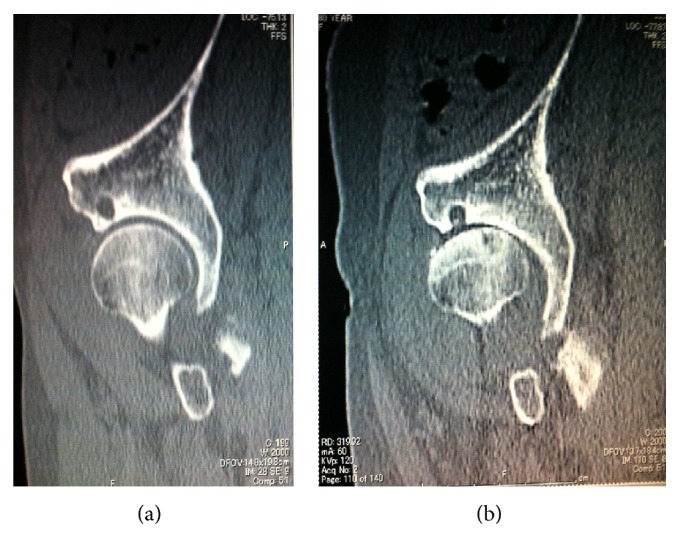
Sagittal plane of CT. Before right THA, enough joint space with the bone cyst at anterior part of the acetabulum (a). Before Left THA, narrowing joint space with destruction of the bone cyst at anterior part of the acetabulum. Femoral head is also flattened with subchondral sclerotic changes due to osteoarthritis development (b).

**Figure 3:**
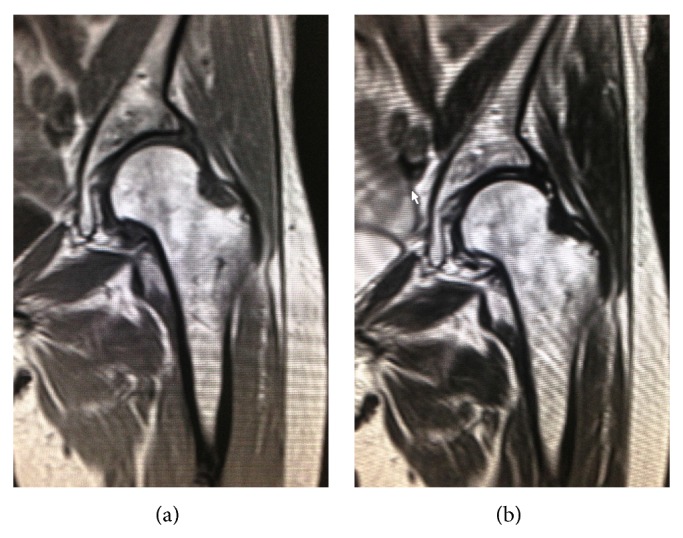
MRI of T1 weighted image (a) and T2 weight image (b) showed no abnormality of the left hip.

Concerning the radiographic evaluation, standard X-ray of the hip and CT scan were obtained. At the time of right THA, left hip was showing enough joint space with aectabular cyst at anterior part (Figures 1(a) and 1(b)). CT scan revealed the bone cyst at the anterolateral part of the acetabulum (Figure 2(a)). After right THA, the joint space at left hip was progressively narrowed (Figure 1(d)), and CT scan showed bony destruction of the acetabulum and femoral head, and the destruction was mostly marked around the acetabular cyst (Figure 2(b)). MRI at the right THA did not show any abnormality in left hip (Figure 3).

**Figure 4:**
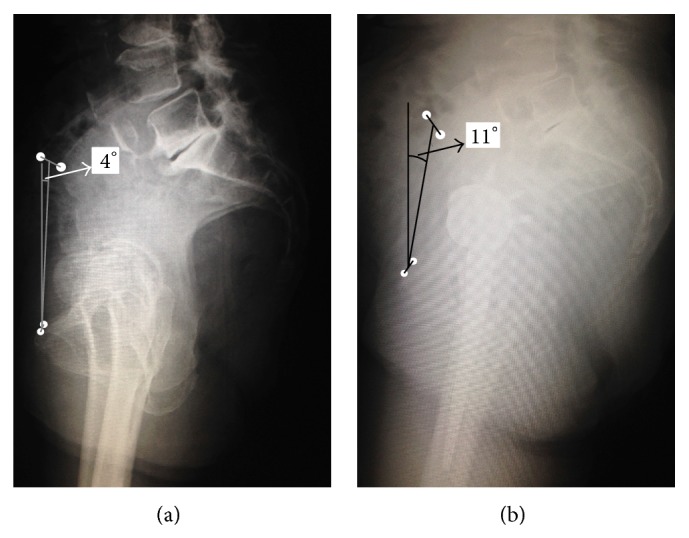
Alignment change assessment using the standing lateral X-ray. The pelvic tilt was defined as the angle between the vertical line and the anterior pelvic plain, which was constructed by the line passing through the midpoint of the bilateral anterior iliac spines and the midpoint of the bilateral pubic tubercles. At the time of right THA (a), the pelvic tilt angle was 4°. At the time of left THA, the pelvic tilt angle was 11°. The pelvic was tilted posteriorly about 6°.

For sagittal alignment assessment, full-length standing lateral radiograph was used; in brief the subject stood in a relaxed position, with the arms folded across the chest to minimize variation due to the effects of trunk posture on the lumbosacral junction [5]. The pelvic tilt was defined as the angle between the vertical line and the anterior pelvic plain, which was constructed by the line passing through the midpoint of the bilateral anterior iliac spines and the midpoint of the bilateral pubic tubercles [6]. Before left THA, the pelvic gained about 6 degrees posterior pelvic tilt compared to before right THA (Figures 4(a) and 4(b)).

Left THA was performed under general anesthesia. The intra-articular joint fluid was sent for the microorganisms examination (the standard culture revealed negative). After left THA, no complication was observed.

## 3. Discussion 

Postel and Kerboull firstly described RDC in English literature in 1970 [1]. RDC is considered rare and develops unusual clinical course. It is characterized by rapid joint destruction (within 6–12 months), and disappearance of the joint space is the typical initial finding on radiographs, followed by rapid disappearance of the femoral head. The incidence has been reported about 1–5% of all THA [7].

Recent studies suggest multiple possible mechanism of the development of RDC. Yamamoto and Bullough reported that SIF of the femoral head occurs in elderly women with osteopenia, and they speculated that insufficiency fracture resulting from osteopenia might lead to rapid breakdown of the hip joint [4]. The term RDC is now only used in cases in which there is no identifiable etiology such as SIF [4], aseptic osteonecrosis [8], intraarticular crystal deposition [9], and articular chondrocalcinosis [10]. After excluding those known pathomechanisms, it is believed that there is unknown other etiologies in RDC. Ogawa et al. revealed that mature and activated osteoclasts exist only in the synovium of RDC and not in OA synovium [2]. They suggest that underlying mechanism of RDC might associate with osteoclastogenesis in the synovium. However, the difficulty of studying RDC is that the data before the onset of RDC is usually unavailable, thus it is unclear whether the findings are the cause or result of the RDC.

**Figure 5:**
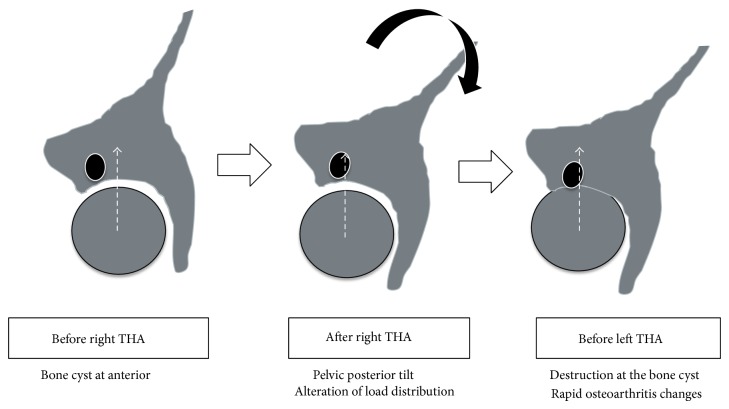
Schema of the estimated mechanism of rapid osteoarthritis changes in our case.

In this report, we presented the patient who had rapid OA development, which meets the current criteria of RDC after contralateral THA. We thought that the increased posterior tilt of the pelvis after THA reinforced the stress concentration at pre-existed anterior acetabular cyst, thereby the destruction of the cyst was occurred. As a result the rapid OA was developed (Figure 5).

In general, pelvic posterior tilt increases according to the ages due to the decrease of the lumbar lordosis and back muscle weakness [11, 12]. By this natural sagittal alignment changes and the fact that the patient with painful hip osteoarthritis tends to flex the hip due to the pain in phase with anterior pelvic tilt, the patient with THA has the pelvic posterior tilt after the operation [13]. In the prosthetic hip with increased posterior pelvic tilt, excessive wear due to edge loading and anterior dislocation might be a clinical problem [14]. On the other hand, in the natural hip with increased pelvic tilt, the acetabular coverage for the femoral head is diminished; subsequently the load distribution on the cartilages is altered, finally leading to decreasing weight bearing area and increasing hip stress [15–17]. This is paradoxically explained by the effect of joint preserving acetabular osteotomy procedure. The principle of acetabular osteotomy is applying the coverage of the femoral head leading to decreasing hip stress and increasing the weight bearing area [18].

Concerning the bone cyst, even though many studies have been performed on the mechanism of development of the bone cyst, the exact mechanism is still not clear. There is the hydrodynamic theory [19], namely, pressure-induced intrusion of joint fluid may cause the enlargement of the cyst, and the mechanical overload theory [20], namely, mechanical stress due to roughness of the articular cartilages causes the cyst. Inui et al. [21] reported that acetabulum cyst exists in the patient with normal joint space, and it arises almost always at anterior part. Whichever the development mechanism of the cyst is, as a consequence, it is obvious that the area of bone cyst is mechanically more fragile compared to sclerotic area. And it is easy to think that the destruction of cyst may occur after the greater load concentration translates at the cyst. Although we guess that the coexistence of the anterior cyst and posterior pelvic tilt influenced to the rapid osteoarthritis change, the presence of the cyst itself could be other explanation, because the base of bone below the cyst is very thin. After the patient underwent the surgery on her right hip, patient might have more activity with higher loading on her left hip. The thin bony barrier collapsed as shown in Figure 2; consequently the lateral coverage of the femoral head was considerably diminished and decreased the weight wearing area and increased hip stress [22].

We think that there is the case of rapid osteoarthritis developing due to alternating load concentration by posterior pelvic tilt on preexisting anterior acetabular cyst such as our patient among the cases diagnosed as RDC without any identifiable etiology. The recognition of sagittal alignment changes and anterior acetabular cyst may play important role in prediction and prevention of the rapid hip osteoarthritis development similar to RDC.
